# Fine structural human phantom in dentistry and instance tooth segmentation

**DOI:** 10.1038/s41598-024-63319-x

**Published:** 2024-06-02

**Authors:** Atsushi Takeya, Keiichiro Watanabe, Akihiro Haga

**Affiliations:** Graduate School of Biomedical Sciences, Tokushima University, 3-18-15 Kuramoto-cho, Tokushima, 770-8503 Japan

**Keywords:** Medical research, Computational biophysics, Dentistry

## Abstract

In this study, we present the development of a fine structural human phantom designed specifically for applications in dentistry. This research focused on assessing the viability of applying medical computer vision techniques to the task of segmenting individual teeth within a phantom. Using a virtual cone-beam computed tomography (CBCT) system, we generated over 170,000 training datasets. These datasets were produced by varying the elemental densities and tooth sizes within the human phantom, as well as varying the X-ray spectrum, noise intensity, and projection cutoff intensity in the virtual CBCT system. The deep-learning (DL) based tooth segmentation model was trained using the generated datasets. The results demonstrate an agreement with manual contouring when applied to clinical CBCT data. Specifically, the Dice similarity coefficient exceeded 0.87, indicating the robust performance of the developed segmentation model even when virtual imaging was used. The present results show the practical utility of virtual imaging techniques in dentistry and highlight the potential of medical computer vision for enhancing precision and efficiency in dental imaging processes.

## Introduction

Oral health is a sensitive indicator of overall health and therefore, developing better dental treatment plays an important role in increasing quality of life. In clinical dentistry, medical imaging with different modalities, such, as two-dimensional (2D) panoramic X-rays^1^, three dimensional (3D) intraoral scans^2,3^, and 3D cone-beam computed tomography (3D CBCT) images^4,5^, is frequently used to assist in diagnosis and/or treatment planning. Among these, in particular, the CBCT can provide fine-structural 3D volumetric information, including that of tooth roots and alveolar bone^6–8^. The segmentation of each tooth based on the CBCT imaging could be used to generate a precisely reconstructed 3D model using computer-aided design with a 3D printer^9–12^. Several studies have attempted to conduct automatic or semiautomatic tooth detection and segmentation based on the CBCT images^13,14^. Owing to the heterogeneous intensity distribution, unclear boundaries between the tooth root and alveolar bone, and large variations in their geometry, conventional segmentation approaches require laborious manual correction to achieve accurate segmentation, which is time-consuming and results in interoperator variability problems. Despite these efforts, accurate segmentation is still a challenging task.

Recently, deep-learning (DL) methods based on convolutional neural networks (CNNs) have been investigated in the field of dentistry for overcoming the limitations associated with conventional segmentation methods^15–20^. Based on CBCT images, DL has allowed the location of teeth to be detected, and thus, segmentation can be performed precisely. Several previous studies have shown the usefulness of DL methods, and consistent evidence has accumulated^21^. However, DL approaches have common problems in their clinical application. First, DL methods require a large dataset for training. Training data are sometimes limited, especially, for rare diseases. With respect to instance tooth segmentation, the training data for each tooth region are defined by the human eye, which limits the preparation of training data. The other problem is the creation of the training data. In the clinical data, the meaning of the ground truth is ambiguous. For example, the segmentation model using DL has been trained with manually segmented teeth, which could include interobserver error. Thus, based on the clinical data, it is impossible to avoid the ambiguity of the ground truth. Nevertheless, the DL model is a promising approach to autosegmentation in dentistry due to its strong ability to learn representative and predictive features in a task-oriented fashion from large-scale data.

To overcome such the problems in the DL approach, in this study, we developed a fine-structural human phantom in dentistry. This digital phantom has a size of 1000 × 1000 × 511 with an equally high resolution of 0.2-mm scale and 12 anatomical regions (tooth, cranium cortical, cranium spongiosa, etc.), which were segmented based on 3D CBCT images for adult male. The anatomies are composed of six major elements (H, C, N, O, P, and Ca), and the weight fractions in each anatomy as well as the total density can vary so that the variety of tooth density can be easily enhanced. This type of the virtual human phantom is not new; for example, the International Commission on Radiological Protection (ICRP) 110 adult female/male phantoms were developed to evaluate radiation exposure to improve radiation protection^22^. ICRP 110 phantoms, however, have poor spatial resolution (2.137 × 2.137 × 8 mm^3^ for male and 1, 775 × 1, 775 × 4.84 mm^3^ for female), which is not applicable in dentistry, and to the best of our knowledge, there is no digital phantom with higher resolution applicable in dental segmentation.

With this developed phantom, we also developed a virtual CBCT system^23^ to produce the various 3D CBCT images by sampling the tube voltage for incident X-rays and the noise of the cone-beam projection images on a flat panel detector (FPD). By referring to the typical geometry of a dental CBCT device, the cone-beam X-ray projections passing through the aforementioned digital human phantom are simulated by a material-based forward projection algorithm from the source to each element in an FPD with a size of 2000 × 1000. By acquiring the projections during gantry rotation, CBCT images corresponding to the digital human phantom can be reconstructed. These paired data can be regarded as a training dataset, such as for creating an instance segmentation model using the DL approach.

**Figure 1:**
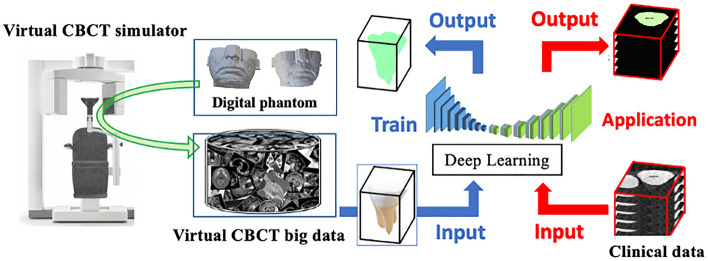
Medical computer vision for instance tooth segmentation.

The conceptual design of this study is depicted in Fig. 1. The proposed approach is completely distinguished from other previously proposed approaches in the context of the DL model in dental segmentation; namely, for DL model training, we generated datasets from virtual systems, not clinical systems. From the machine learning viewpoint, this approach is regarded as a generative model of images. We emphasize here that the errors originating from the manual segmentation that are commonly introduced during the preparation of training data were excluded by means of a feature of the generative model: the CBCT image is directly associated with the ground truth (anatomical information in the virtual phantom).

In this study, our purpose was twofold: one was to develop a fine-structural human phantom in dentistry to create a large-scale virtual CBCT dataset. For this purpose, phantom transformation and various CBCT imaging conditions were introduced. The other objective was to demonstrate the utility of a virtual CBCT dataset in 3D instance tooth segmentation. A DL model was trained with a portion of the data and was validated with the rest to evaluate the model's accuracy. Additionally, clinical CBCT with manually segmented tooth data was applied to assess the feasibility of the use of the virtual system for assessing actual clinical data.

## Results

### Virtual CBCT image

**Figure 2:**
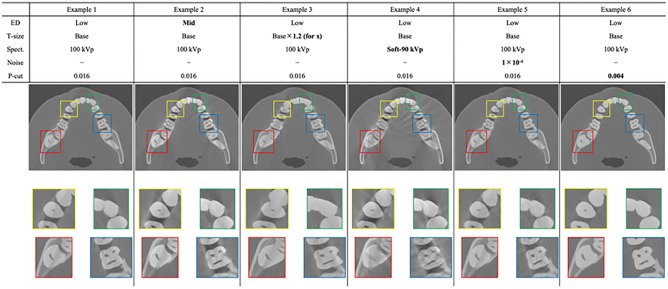
Virtual cone-beam CT generated by computer vision system. The upper table indicates the phantom information and the scanning condition used in the CBCT production, where “ED”, “T-size”, “Spect.”, “Noise”, and “P-cut” indicate elemental density, tooth size, X-ray spectrum, noise intensity, and projection cutoff intensity, respectively. The extended images of four tooth areas surrounded by red, green, blue, and yellow boxes are shown at the bottom.

Various CBCT images were generated under the different scanning conditions, including different tube voltages and noise/cutoff intensities, as well as with different human phantoms expanded from the reference phantoms by tooth deformation and density variation. The representative CBCT images are shown in Fig. 2, where the extended images of the four tooth areas surrounded by red, green, blue, and yellow boxes are also shown at the bottom. The upper table indicates the phantom information and the scanning conditions used in the CBCT, where “ED”, “T-size”, “Spect.”, “Noise”, and “P-cut” indicate the elemental density, tooth size, X-ray spectrum, noise intensity, and projection cutoff intensity, respectively. That is, the reconstructed CBCT images were generated by the corresponding material information/scanning conditions.

The first to third columns show the CBCT images with the base phantom with different material densities from the base, as well as with the deformed tooth in the x-direction, whereas the fourth to sixth columns show the CBCT images with different scanning conditions of tube voltage, noise intensity, and cutoff intensity. The details of the noise/cutoff intensity are described in the “Materials and methods” section. As shown in Fig. 2, the image variety in CBCT can be improved by controlling the scan condition virtually. This indicates that the root causes of nonideal image production in real systems can be investigated via virtual computer vision. In addition, simulations under various conditions also play the role of image generators in medical computer vision. In the next section, the results of the instance tooth segmentation by the DL model trained with the virtual imaging are shown.

### Instance tooth segmentation

**Figure 3:**
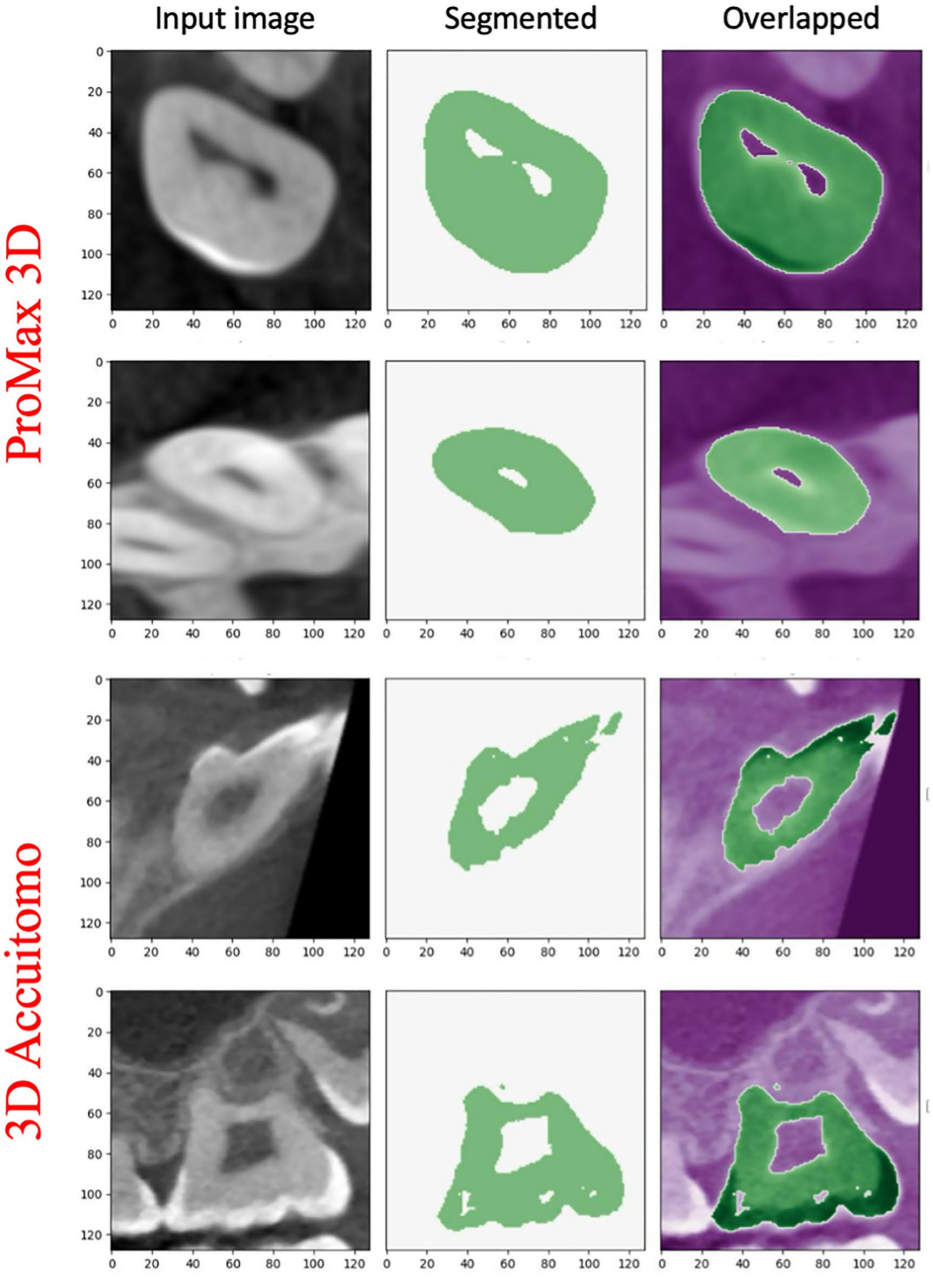
Segmentation by DL model developed with virtual CBCT images. The representative result in the input CBCT acquired by the ProMax 3D scanner and by the 3D Accuitiomo are shown. “True” means manual segmentation, whereas “Pred” means DL segmentation. The difference images between “True” and “Pred” are depicted in the last column.

Virtual CBCT images produced by computer vision include the information on anatomical details. Therefore, one can apply this computer vision system as a generator of training data to reproduce these details from the reconstructed CBCT images. In this study, we developed virtual human phantoms with individual teeth. Using these human phantoms and applying various CBCT scan parameters, the results of instance tooth segmentation by the DL model (*virtual* CBCT model) are demonstrated in this section. Figure 3 shows representative segmented images of test data obtained from actual clinical scans using ProMax 3D (PLANMECA) and 3D Accuitomo (MORITA). It was found that tooth segmentation can be performed very accurately by the DL model. It should be emphasized here that the DL model was trained without the clinical data, only with virtual CBCT images (only 25 virtual teeth and these augmented data were used in the model training. See the Materials and Methods section). Therefore, this suggests that the DL model can accurately learn the features of teeth from virtual imaging. Figure 3 shows that segmentation can be performed more accurately on the CBCT images acquired by the ProMax 3D scanner than on those acquired by the 3D Accuitomo scanner. This is because the virtual CBCT simulator used in the training of the DL model was constructed based on a ProMax 3D scanner.

For comparison, we additionally developed a DL model based on clinical scanned images using 3D Accuitomo (MORITA) (*clinical* CBCT model) and the corresponding segmented data (111 real teeth and their segmented data). Table 1 shows the Dice similarity coefficient (DSC) for the test data. Even with different scanners, segmentation can be performed very well. We also found that the DSC in CBCT acquired by the same scanner as used in model development was greater than that in the different scanners. Namely, the DL model developed based on the ProMax 3D scanner can provide relatively accurate segmentation in the CBCT input of ProMax 3D rather than that of 3D Accuitomo. This is the same as in the clinical CBCT model.

**Table 1:** Dice similarity coefficient (DSC) with predicted segmentation for test data.

Model/input data	Virtual CBCT ProMax 3D	Clinical CBCT ProMax 3D	Clinical CBCT 3D Accuitomo
Virtual CBCT model (ProMax 3D based model)	0.98 ± 0.01	0.87 ± 0.04	0.89 ± 0.01
Clinical CBCT model 3D Accuitomo based model	0.90 ± 0.02	0.88 ± 0.04	0.96 ± 0.01

The “virtual CBCT model” was developed with virtual CBCT imaging modeled from ProMax 3D (PLANMECA), whereas the “clinical CBCT model” was developed with clinical CBCT using 3D Accuitomo (MORITA).

## Discussion

The present study introduces a novel dataset preparation strategy for tooth segmentation using a medical computer vision system based on a generative model that has gained attention in its application to medical fields. While automatic teeth segmentation using the adaptive thresholding approach is sensitive to the complicated appearances of dental CBCT images (e.g., limited intensity contrast, image noise/artifact, etc.), the DL approach shows promising applications in teeth segmentation due to its strong ability to learn representative and predictive features with annotated data. On the other hand, the requirement of large-scale datasets is one of the limitations in the development of DL models because manual annotation is required to initially segment individual teeth.

Wu et al. developed a region-of-interest-based DENSEASPP-Unet model for instance segmentation by using 12 CBCT scans for training^35^. ToothNet also used 12 CBCT images for network model training^15^. More recently, 4938 CBCT scans were collected within this line, 3172 of which were used for AI model training^36^. The developed model demonstrated its potential as a powerful system to boost clinical workflows in digital dentistry.

Although such a study demonstrates the importance of collecting large-scale datasets in clinical practice, the requirement for manually annotated large-scale data still presents a fundamental problem in DL model training. Our study overcomes this problem by developing a virtual CBCT system with a fine structural human phantom in dentistry. Unlike previous studies on image generation such as those utilizing generative adversarial networks, our approach distinguishes itself by employing a virtual CBCT model with human phantoms. The image production in our approach is no longer confined to a black box; rather, it resides in a physics model generating CT images with input data encompassing CT geometry, energy spectrum, noise amount, etc. The images generated within this virtual space can be applied to predict latent variables in the model. For instance, in this study, a tooth segmentation model was developed using virtual data, demonstrating that the model can produce acceptable contouring data even for clinical CBCT input.

This promising result encourages the expansion of virtual space applications in medical imaging. Anatomical segmentation^24,25^ using virtual CT datasets is a potential extension. The use of virtual imaging presents advantages, such as eliminating the need for patient irradiation in artificial intelligence (AI) database development and reducing annotation costs, including manual contouring for ground truth preparation. The creation of a database using virtual models minimizes uncertainty in annotation. The present study compared AI segmentation results with manual contouring for clinical CBCT data, yielding an agreement of more than 0.87 in DSC. Modifying manual contouring based on actual segmented results may increase its quality, implying the various benefits of virtual imaging.

The application and development of virtual space in the medical field, such as for creating databases for metal artifact reduction in tooth imaging, are anticipated. In fact, the virtual CT imaging technique is useful for understanding all artifact-producing root causes (aliasing by limited projection views, beam hardening, scattering, noise, etc.)^37^. Virtual imaging, combined with metallic models such as orthodontic brackets/wires, dental implants, crowns and bridges, and amalgam fillings can be performed. This capability, in particular, was utilized in the development of a novel artifact reduction method. The phantom can also be moved during virtual CBCT scans by modeling the gantry rotation. Thus, artifacts observed in the CBCT system, such as patient motion artifacts, can be investigated by extending our virtual system.

Although this study demonstrated the utility of virtual imaging, it has several limitations. First, further development will be required, particularly for creating detailed models for use as human phantoms. This study presents a fine structural human phantom in dentistry; however it does not incorporate tooth elements such as enamel, dentin, or neural circuits. In addition, the head (face)-shape variety is insufficient in our library, so additional anatomical (including dental) deformation may aid in generating different types of phantoms. The inclusion of only healthy teeth in the development of our phantom is acknowledged. Modeling of dental diseases will introduce variety to the dataset, enhancing the robustness of the segmentation model. The extension of both human phantom and CBCT models is crucial for advancing virtual hospitals.

## Methods

### Fine structural human phantom in dentistry

**Figure 4:**
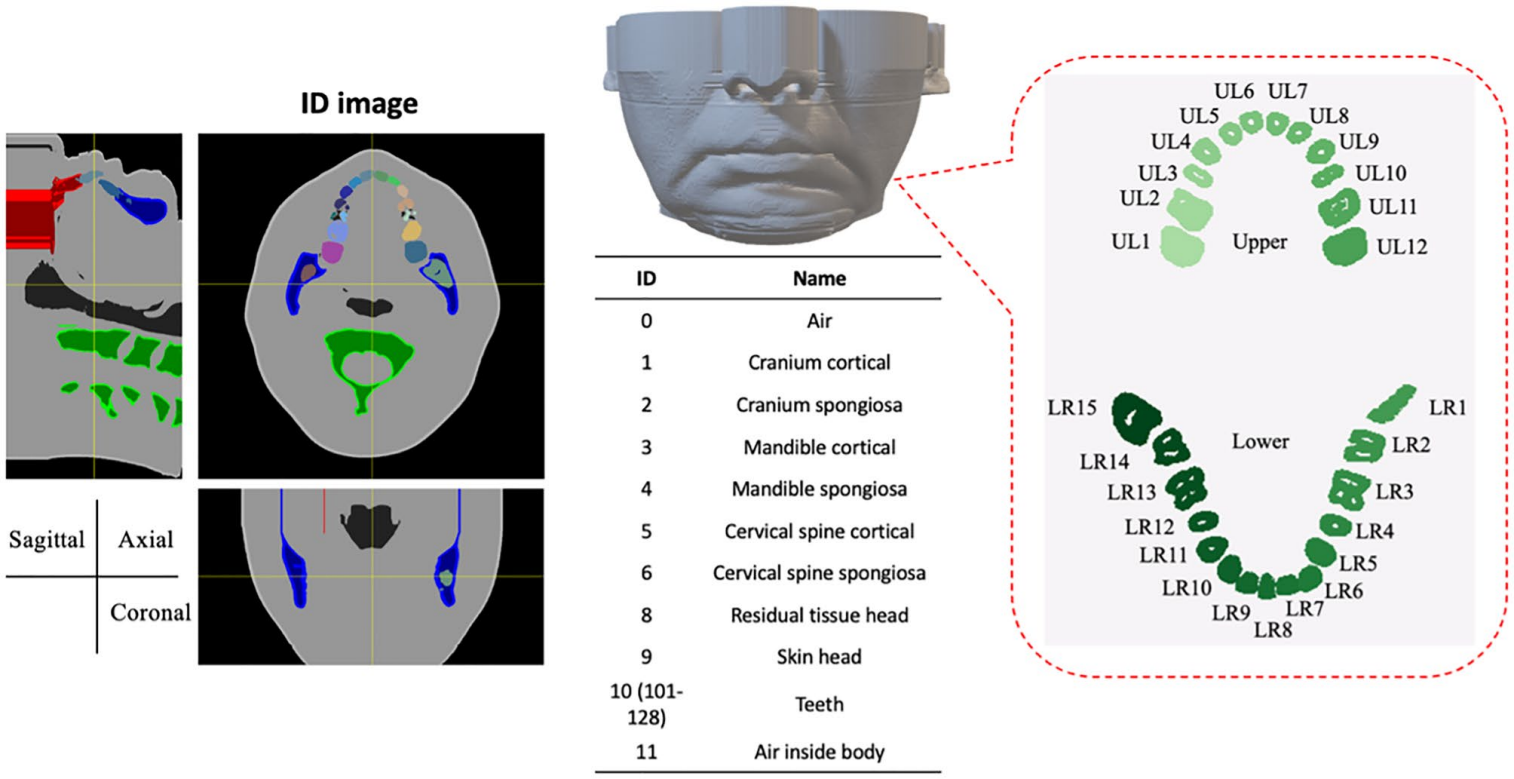
A base phantom in dentistry. Eleven anatomical IDs are assigned for each voxel of jaw region. In addition, IDs are assigned to the individual teeth.

A reference adult human phantom was developed as the base phantom (Fig. 4). This voxel-type human phantom was created by manually enclosing organs based on 3D CBCT images (voxel size: 1001 × 1001 × 512 with resolution: 0.2 × 0.2 × 0.2 cm^3^) obtained in a real clinical setting (ProMax 3D, PLANMECA). Eleven structures (air, cranium cortical, cranium spongiosa, mandible cortical, mandible spongiosa, cervical spine cortical, cervical spine spongiosa, residual tissue head, skin head, teeth, and air inside body) were defined. The human phantom included 12 teeth on top and 15 on the bottom. The upper teeth are referred to as UL1 to UL12 from the left, whereas the lower teeth are referred to as LR1 to LR15 from the right. An anatomical ID is assigned to the individual teeth (101–128). The material density and fraction of the anatomical structures were determined by following the ICRP phantom, as indicated in Table 2. This reference human phantom was used to expand a human library in dentistry by considering the tooth deformation and the deviation of material density in the application of instance tooth segmentation (see the section “Application: instance tooth segmentation”).

**Table 2:** Anatomical structures and corresponding material densities and weights used in this study.

Anatomical structures	ID	ID (ICRP)	ρ (mg/cm^3^)	H (%)	C (%)	N (%)	O (%)	P (%)	Ca (%)
Air	0	0	1	0	0	70	30	0	0
Cranium cortical	1	26	1920	3.6	15.9	4.2	44.8	9.4	21.3
Cranium spongiosa	2	27	1245	8.1	31.7	2.8	45.1	3.7	7.8
Mandible cortical	3	39	1920	3.6	15.9	4.2	44.8	9.4	21.3
Mandible spongiosa	4	40	1189	8.7	35.7	2.6	42.9	3.0	6.3
Cervical spine cortical	5	47	1920	3.6	15.9	4.2	44.8	9.4	21.3
Cervical spine spongiosa	6	48	1135	9.2	35.1	2.9	45.8	2.1	4.3
Residual tissue head	8	116	950	11.4	58.9	0.7	28.7	0	0
Skin head	9	122	1090	10	19.9	4.2	65	0.1	0
Tooth	10 (101–128)	128	2750	2.2	9.5	2.9	42.1	13.7	28.9
Air inside body	11	140	1	0	0	70	30	0	0

The human tissues are composed of 6 elements (H, C, N, O, P, and Ca). The ID (ICRP) is the ID defined in the ICRP computational human phantom^22^. The elemental fractions were followed by the ICRP, whereas three different densities were employed in the bone/tooth regions.

### Virtual computer vision: dental CBCT simulator

The dental CBCT image was produced by virtually scanning the aforementioned human phantom. In this study, the CBCT machine geometry was obtained from ProMax 3D (PLANMECA), where the source-to-isocenter and the source-to-detector distances were set as 30 cm and 55 cm, respectively. The flat panel detector (FPD) was set to a size of 2000 × 1000 pixels, with a 0.2-mm scale in the virtual system. Direct X-rays on the FPD were simulated by the material based forward projection algorithm (MBFPA)^26,27^:

1 $$\frac{{I}_{i}}{{I}_{0}} = \frac{\sum_{E}\alpha (E){n}_{0}(E){e}^{\sum_{j}-{a}_{ij}{\mu }_{j}(E)}}{\sum_{E}\alpha (E){n}_{0}(E)},$$

where $E$ is the photon energy, $\alpha (E)$ is the fraction of the corresponding photon energy bin, ${n}_{0}$ is the photon number in the X-ray source, and ${a}_{ij}$ is the photon pass length in voxel $j$ of the object. The energy bin was set as 1 keV. In this study, two spectra of 90 kVp including 2.5 mm and 6.5 mm Al filters (denoted as soft-90 kVp and hard-90-kVp, respectively), and one spectrum of 100 kVp including a 6.5 mm Al filter, both generated by SPEKTR3.0^28^, were employed. The attenuation ${\mu }_{j}\left(E\right)$ in the ${j}$th voxel can be expressed as the sum of the attenuation coefficients for each element $m$ included in an object:

2 $${\mu }_{j}(E)=\sum_{m}{w}_{m}{\mu }_{m,j}(E,Z,\rho ),$$

${w}_{m}$ is the weight (fraction) of the ${m}$th element and for the human phantoms, $m\in$ H, C, N, O, P, and Ca were considered. For the energy range considered in this study, the linear attenuation coefficient ${\mu }_{m,j}(E,Z,\rho )$ can be written as the sum of the processes of the photoelectric effect, Compton scattering, and Rayleigh scattering as follows:

3 $${\mu }_{m,j}(E,Z,\rho )=\rho Z\frac{{N}_{A}}{A}[{\sigma }_{pe}(E,Z)+{\sigma }_{Comp}(E,Z){ + \sigma }_{Rayl}(E,Z)],$$

where ${N}_{A}$ and $A$ are the Avogadro constant and atomic weight, respectively, and ${\sigma }_{pe}, {\sigma }_{Comp},$ and ${\sigma }_{Rayl}$ are the cross sections owing to the photoelectric effect, Compton scattering, and Rayleigh scattering, respectively, which were obtained from the open X-ray database^29^.

The projection data including noise, ${{I}_{i}}^{*}$, are produced by sampling from the normal distribution $z$:

4 $${{I}_{i}}^{*}={I}_{i}+z\sqrt{{I}_{i}},$$

where ${I}_{i}={I}_{0}{e}^{-{y}_{i}}$ is the X-ray intensity generated in the $i$th detector of a virtual CT. The amount of noise is controlled by ${I}_{0}$, and in this study, it was set to ${I}_{0}\in \{{10}^{4}{,10}^{5}\}$, where typically ${10}^{5}$ provides the same order of the signal-to-noise ratio (SNR) as that observed in a real CT system.

The detector response depends on the photon energy^30^, resulting in a limited dynamic range of the detector. Instead of modeling the detector response, in this study, the cutoff intensity was used to reflect the response on the reconstructed images. That is, the minimum value of the intensity ratio ${I}_{i}$/${I}_{0}$ was set as $c$ and projection data with a value less than $c$ were replaced by $c$. Cutoff intensities $c=$ 0.004 and 0.016 were used in this study.

CBCT images were reconstructed using the Feldkamp–Davis–Kress (FDK) method^31^ with 360 projections for 1° intervals, where the reconstructed sizes were 1000 × 1000 × 350 voxels for each machine parameter and phantom with 0.2 × 0.2 × 0.2 mm^3^ resolution. The reconstruction filter for divergent cone beams proposed by S. Webb was employed (the parameters, $\alpha =0.54, \gamma =1.0, W=0.8$, were used in this study)^32^. Finally, a 3 × 3 median filter was convolved into the reconstructed image to reduce the view aliasing caused by the limited projections. Some of the resulting images are depicted in Fig. 2.

### Application: instance tooth segmentation

**Figure 5:**
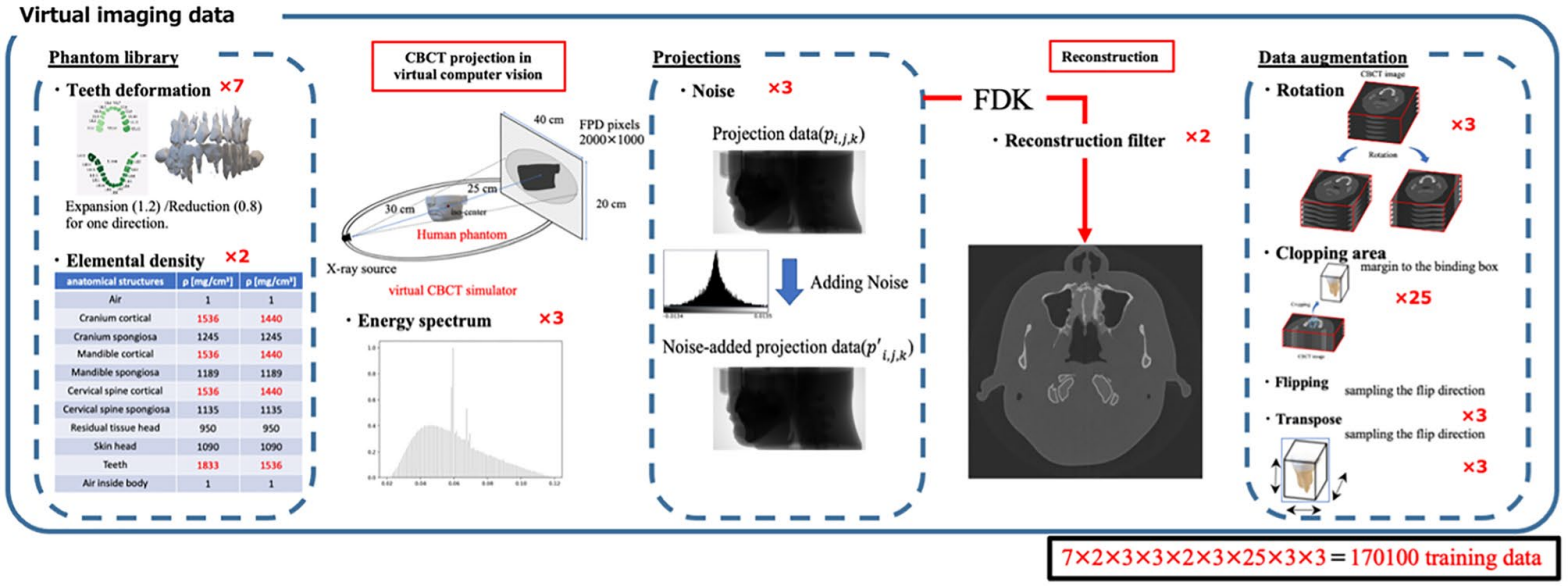
Instance tooth database created in this study. A total of 170,100 teeth were created.

Virtually generated CBCT images were used to develop the DL model for instance tooth segmentation. Figure 5 summarizes how the instance tooth database was created in this study. First, human phantoms were extended by expanding and contracting each tooth in each specific direction so that seven human phantoms in dentistry were prepared. Then, two sets of elemental densities were assigned to the bone-tooth anatomies to create the variety. The virtual CBCT scans were performed with three different energy spectra (soft- and hard-90 kVp and 100 kVp) and with three different noise intensities (no noise, ${I}_{0}\in \{{10}^{4}{,10}^{5}\}$). Each tooth was then cropped three-dimensionally from the generated CBCTs, and these were resliced to 128 × 128 × 128 in voxel size, which included random margins from the binding box of the tooth’s ROI. After flipping and transposing the 3D tooth, a total of 170,100 datasets were created.

A 3D U-net-based DL model for tooth segmentation was developed^33,34^, where the input was a 3D tooth image of 128 × 128 × 128 voxels. Through downsampling, 3 × 3 × 3 convolution with a 2 × 2 × 2 stride was applied for each level of the encoding path. Furthermore, each convolutional layer was batch-normalized and activated by ReLU, except for the downsampling layers, where LeakyReLU was used after batch normalization. Upsampling and skip connection were subsequently performed to output a single binary image corresponding to the segmentation. In the expansive path, 2 × 2 × 2 nearest neighbor upsampling was designed to restore the spatial resolution, similar to the stride convolution in the contracting path.

During training, 500 out of 170,100 images were randomly sampled. Then, 80% of the 500 images were used as the training data and the remaining 20% of the 500 images were used for model selection. The selected model was further tuned by the training data, which were newly sampled from 170,100 images. The optimization was performed with approximately 50 epochs in each step and approximately 2000 epochs in total.

The model evaluation was performed with (1) virtual image datasets, which were excluded from the model development (two teeth), (2) real CBCT images acquired by ProMax 3D (two teeth), and (3) real CBCT images acquired by 3D Accuitomo (two teeth). The cropped area varied among all of the teeth, and the mean DSC and the standard deviation were derived, as shown in Table 1, which indicates that highly accurate segmentation is achievable even if we only use virtual images in the training data.

## Data Availability

The source code of our virtual CBCT simulator is available at: https://github.com/hagaakihiro/VirtualCBCT. The fine structural human phantom in dentistry we developed in this study is found at: https://github.com/hagaakihiro/Dentistry.
